# Increased cardiac index attenuates septic acute kidney injury: a prospective observational study

**DOI:** 10.1186/s12871-015-0005-0

**Published:** 2015-03-01

**Authors:** Jing-chao Luo, Xiao-hua Qiu, Chun Pan, Jian-feng Xie, Tao Yu, Lin Liu, Yi Yang, Hai-bo Qiu

**Affiliations:** Department of Critical Care Medicine, Zhong-Da Hospital, Southeast University School of Medicine, Nanjing, China

**Keywords:** Septic shock, Cardiac index, Acute kidney injury

## Abstract

**Background:**

The relationship between cardiac output and septic acute kidney injury (AKI) remains unclear. The purpose of this study was to assess the association between the cardiac index (CI) and the renal outcomes in patients with septic shock.

**Methods:**

A one-year prospective cohort study was performed in the surgical and medical ICU of a teaching hospital in Nanjing, China. Twenty-nine septic shock patients who required early goal-directed fluid resuscitation were consecutively included. Pulse indicator continuous cardiac output (PiCCO) device was used to measure hemodynamic parameters before and after early goal-directed therapy (EGDT). Based on CI changes after EGDT, patients were assign to the CI increased group or the CI constant group, respectively. The incidence of poor renal outcome, which was defined as AKI on admission without recovery in following three days or new onset AKI within 28 days, was recorded. We investigated whether an increased CI was associated with a better renal outcome.

**Results:**

After EGDT, there were 16 patients in the CI increased group and 13 patients in the CI constant group. The incidence of poor renal outcome was lower in CI increased group than in the CI constant group (6% vs. 62%; P = 0.003) with a relative risk of 0.10. The logistic regression showed that the CI percent change was associated with renal outcome, with an odd ratio of 0.003 (P = 0.056) after adjustment of possible confounding factors. The CI percent change would predict a good renal outcome (AU ROC 0.739, P = 0.012) with moderate accuracy (sensitivity 75% and specificity 89%) when using a 10% cut-off value from Youden index. The CI percent change was also positively correlated with creatinine clearance (CCr) after EGDT (ρ = 0.548; P = 0.002).

**Conclusions:**

The increased CI after EGDT was a protective factor for kidney in patients with septic shock. A CI increased above 10% could be potentially used to predict development and reversibility of AKI in septic shock patients.

**Trial registration:**

Clinicaltrials.gov:NCT01862588 (May 13, 2013).

## Background

As a common comorbidity among septic shock patients, acute kidney injury (AKI) prolongs hospitalization and increases mortality [1-4]. Due to compensatory renal vasoconstriction and right shifting of renal blood flow auto-regulation curve, the renal hypo-perfusion becomes the leading cause of septic AKI [5,6]. Since the organ perfusion was proven to be affected by systemic hemodynamics, early goal-directed therapy (EGDT), which aims to correct circulatory failure [7], may prevent AKI.

Kidney perfusion could be improved through increasing the mean arterial press (MAP) by implement of vasopressors or fluid infusion. Vasopressors may, however, increase the renal vascular resistance, which result in a decrease in renal blood flow [8], making it difficult to set a MAP goal to attenuate AKI [9,10]. In addition, achieving a high MAP goal was related to fluid overload, which may increase the risk of AKI [11,12].

Hyper-dynamic circulation was usually characterized as a high cardiac output. It was more a sign of cardiac compensation, which may lead to a better blood supply in septic shock [13,14]. An increase in cardiac output can be interpreted as an improvement in perfusion, and is associated with restoration of renal blood flow. [14,15]. Moreover, retrospective studies had shown that patients with AKI shared low cardiac index (CI) and higher central venous pressure (CVP) after resuscitation [16,17], suggesting the cardiac output may play an important role during the development of septic AKI.

This prospective cohort study was designed to investigate the relationship between CI and the renal outcomes, with the aim of evaluating the possibility of setting a CI goal for renal protection.

## Methods

### Patients

This non-interventional study was conducted in a 30-bed surgical and medical intensive care unit (ICU) at a teaching hospital affiliated to Southeast University in China. The protocol was approved by Jiangsu institutional Ethics Committee (Approval Number: 2013ZDSYLL075.0), and written informed consent was obtained from patients or their legally authorized representative. The trial was registered at clinicaltrial.gov (NCT01862588).

From January to December 2013, patients admitted into our ICU with documented or suspected septic shock [18] were prospectively screened. Eligibility criteria included a suspected or confirmed infection, two or more criteria for a systemic inflammatory response, and hypotension persisting after initial fluid challenge or blood lactate >4 mmol/L [19]. Exclusion criteria were pregnancy, age <18 years, achieved EGDT goals already on admission, contraindication of invasive catheter, ongoing recovery from AKI, severe chronic renal failure defined by a glomerular-filtration rate (GFR) <30 m/min/1.73 m^2^ [20], as well as conditions known to modify renal perfusion, such as renal-artery stenosis and severe intra-abdominal hypertension (>25 mmHg) [21].

### Study protocol

(1) After admission to the ICU, blood cultures were drawn, antibiotics were administered, and fluid infusion were initiated (2) A pulse indicator continuous cardiac output (PiCCO) device, including internal jugular vein and femoral artery catheters, was placed immediately after getting consent. The hemodynamic parameters were collected through the thermo-dilution method. (3) Due to the observational nature of the study, the physician implemented the EGDT according to the guidelines [19]. (4) After achieving the EGDT goals (CVP 8 to 12 mmHg, MAP ≥ 65 mmHg, urine output (UO) ≥0.5 ml/kg/hr and central venous oxygen saturation (ScvO_2_) ≥ 70% [7]) and allowing patient to stabilize for 30 minutes, the hemodynamic parameters were recorded again. (5) Data collected from patients who achieved EGDT goals within 12 hrs were analyzed, and divided into two groups based on CI changes. Patients were followed up for 28 days, or until death, monitoring the development of AKI.

### Data collection

The basic characteristics such as age, mechanical ventilation, acute physiology and chronic health evaluation score II (APACHE score II), and infection site were collected. Hemodynamic parameters, including CVP, MAP, ScvO_2_, CI, stroke volume index (SVI), systemic vascular resistance index (SVRI), global end-diastolic volume index (GEDI), global ejection fraction (GEF), cardiac function index (CFI), and arterial lactate acid (Lac), were recorded. Additionally, 2-hr urine output, serum creatinine (Cr), serum urea nitrogen (BUN), serum neutrophil gelatinase-associated lipocalin (NGAL, ELISA kit, Uscn Life Science Inc, Wuhan, PRC), urine creatinine, creatinine clearance (CCr) and fractional excretion of sodium (FeNa) were also recorded before and after EGDT. The height and weight were based on recent measurements obtained from patients or their next of kin.

### Outcome measures

The primary end point was poor renal outcome at day 28. For each patient, the incidence of AKI was evaluated according to the Kidney Disease: Improving Global Outcomes (KDIGO) criteria [22], which proposed criteria for three stages of increasing severity. The basal serum creatinine level was set as the lowest value in the 3 months preceding inclusion. If no serum creatinine were available, the lowest value during ICU stay (during renal recovery) or average level of same age bracket among Chinese population [23] was used to assess underlying renal function. Poor renal outcome was defined as persistent AKI (KDIGO stage 2–3 on admission and did not recover to stage 0–1 in 3 days) or development of new AKI (KDIGO stage 2–3) [16,24]. The secondary end point was mortality at day 28.

### Statistics

Data are presented as median and interquartile range or number and percentage, as appropriate. Categorical variables were compared using the Fisher’s exact test, continuous variables were compared using several tests: for pre and post measurements, the nonparametric Wilcoxon test was used; for pairwise comparisons, the Mann–Whitney test; for comparisons across the three group, the Kruskal-wallis test. Receiver operating characteristic (ROC) curves and Youden index were used to choose the appropriate cutoff points. The relationships between variables were evaluated through the use of Spearman correlation. Logistic regression analysis, with or without adjustment for potential confounding factors, was used to confirm the correlation. A significance level 0.05 was chosen for all tests in SPSS 17.0 software.

## Results

### Patients’ characteristics

**Figure 1:**
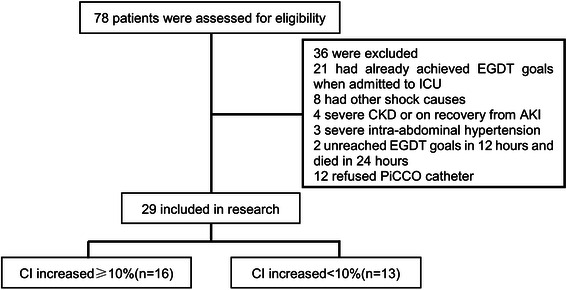
**Flowchart of patients included in the study.** EGDT, early goal-directed therapy; CKD, chronic kidney disease; PiCCO, Pulse indicator continuous cardiac output; CI, cardiac index.

Twenty-nine septic shock patients were included after screening and application of selection criteria (Figure 1). Considering the systemic error of thermo-dilution method, a cut-off value of 10% for CI increase [25] was used. In total, we observed 16 patients with CI increase (CI increased group) and the rest 13 patients were divided in CI constant group. Factors such as age, hypertension or diabetes mellitus, APACHE score II, the use of vasopressors or resuscitation fluid, 28-day mortality, etc. made no significant difference between both groups (Table 1). Yet, the levels of CI, SVI, GEF and CFI after EGDT were higher in the CI constant group than those in the CI increased group (Table 2).

**Table 1:** **Patients’ characteristics**

	CI constant group (n = 13)	CI increased group (n = 16)	P value
Age (years)	78(69–86)	75(65–83)	0.29
Males n (%)	7(54)	8(50)	1.00
Hypertension n (%)	9(69)	10(63)	1.00
Diabetes mellitus n (%)	5(38)	4(25)	0.69
Source of infection			
Lungs n (%)	6(46)	10(63)	0.47
Abdomen n (%)	7(54)	4(25)	0.14
Soft tissue n (%)	0(0)	1(6)	1.00
Other n (%)	0(0)	1(6)	1.00
APACHE score II	24(24–30)	23(19–27)	0.13
SOFA score	11(10–12)	11(8–12)	0.19
Mechanical ventilation n (%)	10(77)	13(81)	0.70
Norepinephrine (ug/kg/min)	0.3(0.1-0.4)	0.2(0.2-0.4)	0.36
Fluid Therapy			
Hydroxyethyl starch n (%)	8(62)	7(44)	0.46
Colloid fluid n (%)	11(85)	13(81)	1.00
Hydroxyethyl starch amount (ml/kg)	11.7(7.1-18.6)	7.9(7.1-10.0)	0.20
Colloid fluid amount (ml/kg)	13.8(7.1-20.0)	8.2(5.0-10.6)	0.20
Crystalloid fluid amount (ml/kg)	26.8(15.7-34.7)	28.3(18.9-42.8)	0.51
Resuscitation fluid amount(ml/kg)	35.8(27.6-53.6)	36.1(25.8-52.4)	0.86
EGDT period (hour)	6.0(6.0 -7.0)	6.0(5.0-6.0)	0.27
Poor renal outcome n (%)	8(62)	1(6)	0.003
AKI 2-3on admission n (%)	6(46)	4(25)	0.27
Persistent AKI n (%)	4(31)	1(6)	0.14
AKI 0-1on admission n (%)	7(54)	12(75)	0.27
New AKI n (%)	2(15)	0(0)	0.19
Use of renal-replacement therapy n (%)	6(46)	3(19)	0.23
Days in ICU (days)	8(6–14)	8(7–19)	0.98
28-day mortality n (%)	6(46)	5(31)	0.47

CI, cardiac index; APACHE, Acute physiology, age, chronic health evaluation; SOFA, sequential organ failure assessment. The data in the table are expressed as median (interquartile range) or number (%).

**Table 2:** **Hemodynamic parameters before and after EGDT**

		CI constant group (n = 13)	CI increased group (n = 16)	P value
HR (beats/min)	Before EGDT	113(90–134)	117(87–130)	0.69
	After EGDT	91(73–114)	95(78–107)	0.76
	P value	0.002	0.013	
MAP (mmHg)	Before EGDT	65(58–73)	64(55–67)	0.60
	After EGDT	87(69–98)	86(76–93)	0.96
	P value	0.002	<0.001	
CVP (mmHg)	Before EGDT	10(5–12)	6(4–11)	0.56
	After EGDT	10(8–15)	11(8–15)	0.89
	P value	0.045	0.004	
CI (L/min/m^2^)	Before EGDT	3.2(2.7–4.3)	2.6(2.2–3.2)	0.06
	After EGDT	3.0(2.6–4.0)	3.8(3.1–5.2)	0.028
	P value	0.022	<0.001	
SVI (ml/m^2^)	Before EGDT	34(28–37)	29(23–36)	0.14
	After EGDT	36(32–38)	43(40– 48)	0.003
	P value	0.11	<0.001	
SVRI (dyn.s.m^2^/cm^5^)	Before EGDT	1478(1231–2076)	1722(1497–2373)	0.34
	After EGDT	1652(1273–2391)	1545(1185–1881)	0.43
	P value	0.06	0.163	
GEDI (ml/m^2^)	Before EGDT	793(670–936)	651(634–771)	0.035
	After EGDT	878(673–1056)	767(697–886)	0.50
	P value	0.17	<0.001	
GEF (%)	Before EGDT	15(13–20)	17(16–22)	0.19
	After EGDT	16(13–20)	23(20–24)	0.012
	P value	0.36	0.013	
CFI (L/min)	Before EGDT	4.0(3.2–5.3)	4.2(3.7–4.9)	0.96
	After EGDT	3.8(2.9–4.5)	5.0(4.4–5.9)	0.003
	P value	0.008	0.003	
ScvO_2_ (%)	Before EGDT	80(73–85)	79(76–81)	0.66
	After EGDT	80(75–83)	84(81–84)	0.026
	P value	0.81	0.005	
Lac (mmol/L)	Before EGDT	3.2(1.5–5.1)	2.4(1.3–3.3)	0.44
	After EGDT	1.7(1.2–2.8)	1.3(0.9–1.7)	0.25
	P value	0.039	0.03	

MAP, mean arterial pressure; CVP, central venous pressure; HR, heart rate; CI, cardiac index; SVI, stroke volume index; SVRI, systemic vascular resistance index; GEDI, global end-diastolic volume index; GEF, global ejection fraction; ScvO2, central venous oxygen saturation; Lac, arterial lactate acid. The data in the table are expressed as median (interquartile range) or number (%).

### Relationship between CI changes and renal function during resuscitation

**Figure 2:**
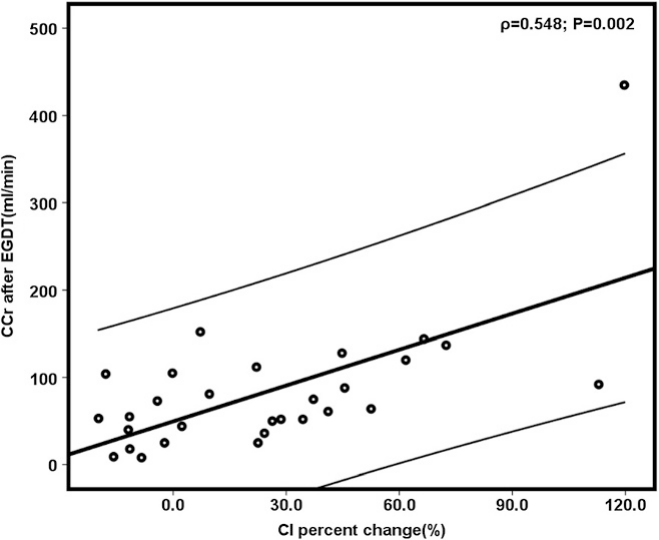
**The relationship between cardiac index percent change and creatinine clearance after resuscitation.** Correlations were assessed by using Spearman regression model. Figure 2 shows a significant positive correlation between CI percent change and Ccr after resuscitation (ρ = 0.548; P = 0.002).

After EGDT, the urine output and CCr increased in CI increased group while decreased in CI constant group (Table 3). The median CCr after resuscitation was higher in CI increased group than in CI constant group (Table 3). A linear regression model shown that the CI percent change associated with CCr percent change (ρ = 0.524; P = 0.004) and CCr level after resuscitation (ρ = 0.548; P = 0.002) (Figure 2).

**Table 3:** **Renal function parameters before and after EGDT**

		CI constant group (n = 13)	CI increased group (n = 16)	P value
Serum Cr(umol/L)	Before EGDT	68(49–261)	83(56–133)	0.74
	After EGDT	83(55–215)	76(63–127)	0.86
	P value	1.0	0.98	
Serum Bun(mmol/L)	Before EGDT	8.4(4.9-14.4)	7.8(5.7-10.0)	0.61
	After EGDT	8.5(4.5-14.8)	7.9(4.7-9.0)	0.65
	P value	0.27	0.27	
Serum NGAL(ng/ml)	Before EGDT	140(70–375)	74(28–148)	0.11
	After EGDT	169(60–254)	125(60–179)	0.55
	P value	0.68	0.048	
Urinary Cr(umol/L)	Before EGDT	4010 (2395–9143)	7894(3424–9910)	0.24
	After EGDT	2766(2100–8817)	3642(2174–7375)	0.69
	P value	0.60	0.004	
Urine output(ml/h)	Before EGDT	85(40–128)	73(23–119)	0.35
	After EGDT	67(43–128)	98(70–150)	0.11
	P value	0.31	0.013	
Ccr (ml/min)	Before EGDT	85(17–129)	59(45–101)	0.69
	After EGDT	53(22–93)	82 (52–126)	0.08
	P value	0.034	0.12	
FeNa(%)	Before EGDT	0.6(0.1-12.3)	0.7(0.3-1.5)	0.64
	After EGDT	1.0(0.1-8.9)	1.0(0.5-1.8)	0.83
	P value	0.53	0.41	

Cr, creatinine; Bun, urea nitrogen; NGAL, neutrophil gelatinase-associated lipocalin; Ccr, creatinine clearance; FeNa, fractional excretion of sodium. The data in the table are expressed as median (interquartile range) or number (%).

### Relationship between CI changes and renal outcomes

**Figure 3:**
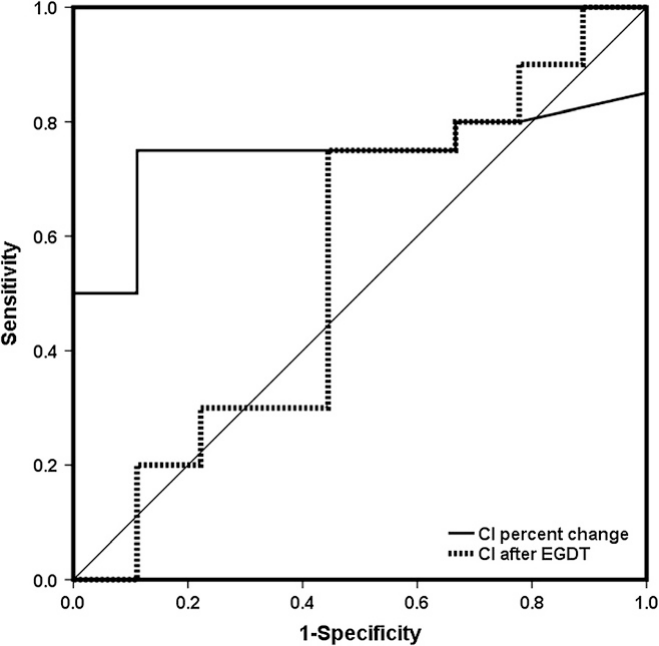
**Receiver-operating characteristic (ROC) curve depicting the cardiac index (CI) percent change (%) or CI after early goal-directed therapy (EGDT) to predict good renal outcome.** The area under ROC curves were 0.739(P = 0.012) of CI percent change to predict non-poor renal outcome. A cutoff value of 10% determined by Youden index would provide a sensitivity of 89% and a specificity of 75%. The area under ROC curves were 0.556(P = 0.115) of CI after EGDT to predict non-poor renal outcome. A cutoff value of 3.0 L/min/m^2^ determined by Youden index would provide a sensitivity of 56% and a specificity of 75%.

The incidence of poor renal outcomes was lower in CI increased group than in the CI constant group (6% vs. 62%; P = 0.003) with a relative risk of 0.10. The single-factor analysis also shown that the CI percent change (33% [2% to 59%] vs. 0 [−10% to 8%]; P = 0.043) differed between patients with different renal outcomes. After adjustment of possible confounding factors (GEDI percent change: OR = 1.625, P = 0.910; APACHE score II: OR = 1.067, P = 0.598; total resuscitation fluid amount: OR = 1.038, P = 0.365; basal serum Cr level: OR = 0.987, P = 0.229), the OR between CI percent change and poor renal outcome was 0.003 (P = 0.056). The CI percent change had a moderate predictive accuracy of AKI recovery with an area under the ROC curve of 0.739(P = 0.012) (Figure 3). A cutoff value of 10% increased CI determined by the Youden index would reach a sensitivity of 75% and a specificity of 89%.

### Relationship between CI after EGDT and renal outcome

Based on a retrospective study [17] which reported that a much high cardiac output was associated with worse outcome, we divided patients into three groups according to the CI after EGDT. The incidence of poor renal outcomes was 44% (4/9) in patients with a CI < 3.0 L/min/m^2^, 10% (1/10) in patients with a CI 3.0-4.0 L/min/m^2^ and 40% (4/10) in patients with a CI > 4.0 L/min/m^2^, however, no statistical differences were observed (P = 0.20) (Table 4). The CI after EGDT was not sufficient to predict renal outcome with an area under the ROC curve of 0.556 (P = 0.115) (Figure 3).

**Table 4:** **Relation between hemodynamic parameters and outcomes**

	CI < 3.0 L/min/m2 (n = 9)	CI 3.0-4.0 L/min/m2 (n = 10)	CI > 4.0 L/min/m2 (n = 10)	P value
Age (years)	81(76–86)	75(63–84)	74(61–86)	0.39
APACHE score II	24(20–25)	24(20–31)	25(20–30)	0.70
CVP after EGDT(mmHg)	8(7–11)	13(10–15)	11(9–16)	0.03
GEDI after EGDT(ml/m^2^)	722(613–970)	785(696–950)	881(766–952)	0.42
MAP after EGDT(mmHg)	95(85–102)	85(76–93)	77(71–90)	0.06
ScvO_2_ after EGDT(%)	82(74–85)	81(80–83)	84(82–87)	0.17
Norepinephrine (ug/kg/min)	0.1(0–0.3)	0.3(0.1-0.7)	0.2 (0.1-0.4)	0.54
Hydroxyethyl starch n (%)	5(56)	6(60)	4(40)	0.98
Hydroxyethyl starch amount (ml/kg)	10.0(7.1-12.2)	9.0(6.3-12.5)	10.6(7.3-18.9)	0.77
Resuscitation fluid amount(ml/kg)	31.7(19.4-37.9)	33.9(25.2-55.9)	44.5(32.3-62.9)	0.08
Poor renal outcome n (%)	4(44)	1(10)	4(40)	0.20
28-day mortality n (%)	4(44)	3(30)	4(40)	0.80

CI, cardiac index; APACHE, Acute physiology; CVP, central venous pressure; GEDI, global end-diastolic volume index; MAP, mean arterial pressure; ScvO_2,_ central venous oxygen saturation; The data in the table are expressed as median (interquartile range) or number (%).

## Discussion

The present study demonstrated that the increased CI during EGDT was a protective factor of AKI. The increased CI was not only associated with better renal function, but also associated with better renal outcome.

Renal hypo-perfusion was one of the main causes of septic AKI [6], thus restoration of renal blood flow may benefit kidney. Hyper-dynamic circulation, characterized by high cardiac output, was common in septic shock and would lead to better perfusion of organs, including heart, brain, liver, guts, and especially the kidney [13-15]. In animal sepsis studies conducted by Langenberg, et al., increased renal blood flow along with cardiac output was also observed [15,26,27]. In this study, despite lacking of direct renal perfusion monitoring, we observed that the CCr was increasing together with CI changes during EGDT, suggesting the kidney may benefit from improvement of global circulation. Furthermore, the renal outcome in CI increased group was better than CI constant group, and a regression analysis has confirmed increased CI was a protective factor for kidney after adjusting any possible confounding factors.

The survival sepsis campaign guidelines well promoted the early initiation of fluid and vasopressors in patients with septic shock. Most patients had already received different doses of fluid before admitted to ICU (in the emergency department or even in the ambulance) and 13 patients in our study complete 3 of the 4 EGDT goals when admitted. However, the EGDT goals, as the fundamental targets, were not the endpoint of early resuscitation [7,28,29]. In this study, the great improvement of MAP, HR, Lac during the early resuscitation were observed, suggesting a potential space for progression, which enabled us to further investigate the benefits of increased CI.

Various reasons might contribute to the constant CI in part of patients. Physiologically, the cardiac output is determined by the volume status and fluid responsiveness. After EGDT, though receiving same dose of fluid, the volume status parameters (CVP or GEDI) increased apparently in CI increased group, suggesting a better fluid responsiveness with these patients. The higher CVP and GEDI in the CI constant group before EGDT indicated a sign of more sufficient blood volume, and further infusion of fluid would not transfer to more cardiac output (Starling principle). Besides, the reduction of myocardial contractility usually results from sepsis, which was induced by myocardial depression [30]. It may also play a role in the absence of fluid responsiveness. We noticed that, concerning the similar volume status (before EGDT in the CI-increased group vs. after EGDT in the CI increased group), the SVI, GEF and CFI were lower in the CI-increased group, and they may show a significant difference in myocardial contractility between groups (Table 2).

Though increased cardiac output may attenuate poor renal outcome, we failed to define a CI goal which has the minimal risk of AKI. Based on a previous research [17], either a much lower or higher cardiac output may be harmful. When divided patients into three groups, the incidence of poor renal outcome distributed in valley form and a CI between 3.0-4.0 L/min/m^2^ seems less likely to develop AKI. There may be two hypotheses for the phenomenon: First, a higher cardiac output not only increases oxygen deliver for the kidney, but also brings harmful inflammatory factors or oxygen free radicals [31]; Second, it is likely that a higher cardiac index is a sign of worse sepsis with poor oxygen extraction and higher compensated perfusion [32], and therefore these patients were more prone to develop AKI. Though it was necessary to evaluate an upper limit of cardiac output goal, however, the area under ROC curve was not good enough to determine a CI goal based on our present data.

Renal protection needs an accurate prediction on development and progression of AKI. However, evaluation for the AKI risk remained uncertainty. Although the AKI biomarker and renal Doppler were two promising methods for AKI evaluation [33,34], they were either too complex or expensive to be widely accepted by clinical physicians and technicians. Our study had demonstrated that the CI changes were correlated with renal outcomes, which a 10% CI increased could be used as an indicator. Besides, the CI could be easily monitored at bedside, which made itself more applicable in critical care departments.

### Limitations

Our study had several limitations. First, being completely blind to the patients’ clinical conditions seemed almost impossible. So a doctor who was unaware of our research details designed the fluid resuscitation plans. Moreover, the sample size was not considerable quantity even though we have adequate power, the study needs to be repeated in a larger, multicenter cohort. Only those patients who had received PiCCO monitor were included in current research, which might introduce bias. Additionally, patients who had received treatment in emergency department and those who had already achieved EGDT goals during ICU admission were excluded, therefore potentially the sample size was reduced. Finally, some patients received infusion of hydroxyethyl starch during resuscitation, which may affect renal function as well. However, there were no significant differences in portions or doses of hydroxyethyl starch between groups.

## Conclusion

In this study, we found that increased CI during EGDT was associated with a better renal outcome and could be potentially used to predict the development or progression of AKI. Targeting a 10% CI increment for reducing the risk of AKI should be considered in this setting.

## Abbreviations

AKI: Acute kidney injury
APACHE score II: Acute physiology and chronic health evaluation score II
BUN: Serum urea nitrogen
CCr: Creatinine clearance
CFI: Cardiac function index
CI: Cardiac index
CVP: Central venous pressure
EGDT: Early goal directed therapy
FeNa: Fractional excretion of sodium
GEDI: Global end-diastolic volume index
GEF: Global ejection fraction
GFR: Glomerular-filtration rate
HR: Heart rate
MAP: Mean arterial pressure
NGAL: Neutrophil gelatinase-associated lipocalin
PiCCO: Pulse indicator continuous cardiac output
ScvO2: Central venous oxygen saturation
SVI: Stroke volume index
SVRI: Systemic vascular resistance index
UO: Urine output
